# Biotechnologies in Perfume Manufacturing: Metabolic Engineering of Terpenoid Biosynthesis

**DOI:** 10.3390/ijms24097874

**Published:** 2023-04-26

**Authors:** Alessia Shelby Manina, Fabio Forlani

**Affiliations:** Department of Food Environmental and Nutritional Science (DeFENS), University of Milan, 20133 Milan, Italy

**Keywords:** terpenes, metabolic engineering, bio-based ingredients, fragrances

## Abstract

The fragrance industry is increasingly turning to biotechnology to produce sustainable and high-quality fragrance ingredients. Microbial-based approaches have been found to be particularly promising, as they offer a more practical, economical and sustainable alternative to plant-based biotechnological methods for producing terpene derivatives of perfumery interest. Among the evaluated works, the heterologous expression of both terpene synthase and mevalonate pathway into *Escherichia coli* has shown the highest yields. Biotechnology solutions have the potential to help address the growing demand for sustainable and high-quality fragrance ingredients in an economically viable and responsible manner. These approaches can help compensate for supply issues of rare or impermanent raw materials, while also meeting the increasing demand for sustainable ingredients and processes. Although scaling up biotransformation processes can present challenges, they also offer advantages in terms of safety and energy savings. Exploring microbial cell factories for the production of natural fragrance compounds is a promising solution to both supply difficulties and the demand for sustainable ingredients and processes in the fragrance industry.

## 1. Introduction

Essential oils, which are aromatic and volatile liquids, are produced from plant material mainly by distillation-based methods and are usually named according to the sourcing plant species [1]. Essential oils can be described as either mixtures of fragrant substances or as mixtures of fragrant and odorless substances [2]. They have always been used for various purposes: in the past, essential oils were mainly used to treat various types of infectious diseases around the world, while nowadays at least 300 types of essential oils out of 3000 are commercially important in various types of industries, including that of fragrances, health care, cosmetics, food, beverages, agronomics, and pharmaceuticals [3]. With regard to the fragrance sector, essential oils can also be described—and to some extent classified—based on their scent [1]. In fact, essential oils that are used in perfumes are generally classified according to their volatility, which is the speed with which they diffuse in the air; based on this characteristic, each essential oil can be identified/classified into one of three “notes”: top notes, heart notes, and base notes.

From a biological point of view, these mixtures of fragrant substances within the plant are mainly composed of secondary metabolites. Secondary metabolites are historically so named to conventionally separate them from those of the energetic and biosynthetic primary metabolism. However, as their main role is to provide an evolutionary advantage to the plant which produces them, they should rather be called specialized metabolites; in fact, these metabolites have different types of biological activities, including antibacterial, antioxidant, antiviral, insecticidal, etc., and can play ecological roles, such as in fire tolerance, attracting pollinators and/or herbivores for seed dispersal, drought tolerance or plant-to-plant biosemiosis (pheromones) [1,4].

From a biochemical point of view, the scent of essential oils is associated with several unique combinations of low molecular weight (below 400 Da) volatile organic compounds [5]. In general, most of the components of essential oils derive from three main classes of compounds—terpenes, phenylpropanoids/benzenoids and fatty acid derivatives—which are often modified (oxidized, esterified, methylated, etc.) altering the volatility of such compounds in the final phases of their formation [6]. Nitrogen- and sulfur-containing compounds may also be present [7]. Amongst these three classes, terpene compounds generally constitute a major part of essential oils. In fact, monoterpenes and sesquiterpenes, a representative group of known natural volatile products [7], are classical constituents of essential oils and exhibit an extremely wide diversity of biological structures and properties. Common scented constituents of essential oil include monoterpenes, such as linalool, geraniol, myrcene, trans-β-ocimene, and limonene. Limonene is a cyclic monoterpene with citrus notes, which is often used as a top note in the production of perfumes and is nowadays mainly obtained from waste derived from orange juice production. The sesquiterpene compounds α- and β-santalol are key scented components of the sandalwood essential oil which is obtained from the heartwood and roots of mature (>25 years) oil-producing Santalaceae (*Santalum* genus) plants via steam distillation [8]. Patchoulol is a scented sesquiterpene compound which accounts for 30–40% of the total mass of compounds contained in patchouli oil, an essential oil commonly obtained from the leaves of *Pogostemon cablin* (the patchouli plant), which is widely used in the perfume industry [9].

Nowadays, most aromatic and scented compounds are produced either through chemical synthesis or extraction from natural materials (such as plant and animal sources). However, extraction from plants has some disadvantages: plant-derived materials are often subjected to fluctuations in price, annual production volumes and quality, due to factors related to seasons, geographical area of production, geopolitical problems, climate disasters and plant diseases. In addition, the price of compounds obtained from plants can increase due to limited cultivation, scarcity of the compounds of interest within the extract, high need of labor for the harvest or the depletion of natural resources [10]. Today, chemical synthesis still represents the most economical technology for the production of most aromatic and scented compounds; however, it can require unsustainable conditions (toxic catalysts, high pressure and temperature) and usually lacks adequate regio- and enantio-selectivity of the substrate, resulting in a mixture of isomers. Furthermore, the generated compounds are labeled as “artificial” or “nature-identical”, which ultimately decreases their economic value [11]. Due to these drawbacks and consumers’ growing interest in natural products and sustainable processes, suitable alternative strategies have been sought in recent years to produce natural flavorings and fragrant compounds, and in particular of terpenes and terpenoids (also known as isoprenoids).

Biotechnology approaches in the fragrance sector have been developing for several decades, with the first research papers on biotechnological production of fragrances being published in the 1980s [12]. However, it was not until the 2000s that these approaches began to gain significant attention. Since then, a multitude of biocatalytic approaches have been developed to enhance the quality and quantity of essential oils and natural extracts used in perfumery [13]. Initially, pre-treatment techniques utilizing cellulases, pectinases, and glycosidases were developed to increase extraction or distillation yields by facilitating the release of volatile molecules from cellular compartments [13]. Recently, the field has grown in complexity and scope, either focusing on cutting-edge techniques such as biocatalysis, metabolic engineering, synthetic biology, gene editing and cloning, and analytical chemistry, or by taking advantage of the new knowledge on biosynthetic pathways. This has led to the development of novel ingredient classes, including biotech ingredients obtained through metabolic engineering, such as bioproduced compounds, either pure or as blends of essential oil components [13]. Recently, the fragrance industry has shifted towards sustainable and eco-friendly production methods and away from animal-derived raw materials. Biotechnology is gaining interest as a new means of extracting fragrance ingredients and is facilitating this transition [14].

In this review, we aim to resume the recent achievements in the production of some of the most relevant terpene compounds used in the fragrance sector via genetic engineering of plants, biotransformation, or de novo synthesis.

## 2. Engineering of the Isoprenoid Pathways

Terpenes are synthesized from five-carbon precursors, isopentenyl diphosphate (IPP) and dimethylallyl diphosphate (DMAPP), which can be derived from two alternative pathways: the mevalonate (MVA) pathway and the non-mevalonate pathway, also known as the 2-*C*-methyl-d-erythritol 4-phosphate (MEP) pathway. While the MVA pathway produces IPP and DMAPP through a series of enzymatic reactions starting from acetyl-CoA, the MEP pathway is found in most bacteria, algae, plants (plastids), and apicomplexan protozoa (e.g., malaria parasites) and it produces IPP and DMAPP from glyceraldehyde 3-phosphate and pyruvate, using a different set of enzymatic reactions [15]. Due to the implication of terpene compounds in many biological functions and their economic value, the MVA and MEP pathways have been extensively studied and important regulatory mechanisms have also been clarified. The condensation of IPP and DMAPP is catalyzed by prenyltransferases, producing the linear prenyl diphosphate precursor for each class of terpenes: geranyl diphosphate (GPP), farnesyl diphosphate (FPP) and geranylgeranyl diphosphate (GGPP) for mono-, sesqui- and diterpenes, respectively. The next step involves the terpene synthases, which are part of a very large family of enzymes; they play a key role in the biosynthesis of terpenes as they catalyze various reactions (e.g., cyclization) on GPP, FPP and GGPP to form the carbon skeletons of terpene compounds and are therefore the key point for the formation of the extremely wide diversity of the final structures [10]. While many terpenes are produced directly from terpene synthases, others are formed through alterations of the initial products by oxidation, dehydrogenation, acylation and other types of reactions [6]. Finally, enzymes, such as monooxygenases of the cytochrome P450 family (P450) and oxidoreductases, are involved in further modifications and decorations of the terpene backbone, producing the thousands of naturally occurring terpenoids [10].

The knowledge accumulated to date regarding the biosynthesis of terpenes opens up various possibilities for the metabolic engineering of all phases of the entire path. In particular, it was found that a key element for the development of biocatalytic pathways is the availability of key genes which lead to the production of the target compounds, especially the genes that code for terpene synthases. Several examples of metabolic engineering for the biosynthesis of terpenes and terpenoids in microorganisms and plants demonstrate the possibilities of developing inexpensive biochemical pathways for the production of terpene compounds which are widely used in the fragrance sector. In fact, one of the most interesting areas of metabolic engineering is focused on the production of natural products in transgenic plants to improve agronomic traits, such as pest resistance and competitiveness, and to alter fragrance and flavor profiles [16].

In plants, two alternative approaches can be used to genetically manipulate the fragrance profile. The first is based on the introduction of foreign genes that encode for enzymes with activities that are lacking in the target plant. The second approach is based on the modulation (down or up-regulation) of the expression of one or more native genes. With the latter approach, the production of a volatile compound can be increased by up-regulating a gene in the pathway or, alternatively, by blocking the production of an unwanted volatile compound [17].

Other interesting methods for the synthesis of aromas and fragrances are based on the use of genetic engineering methods, microbial de novo synthesis (fermentation) or the chemical conversion of natural precursors using biological methods (enzymes or whole cells, and biocatalysis or biotransformation) [18], since the products obtained from this type of processes can be labeled as “natural” [11]. De novo synthesis refers to the production “from the very beginning”, that is, the synthesis of substances starting from simpler substances (sugars, amino acids, nitrogen salts and minerals, among others), which will be metabolized by microorganisms to form diverse and complex structures, generating a mixture of low concentration products [11,14]. Biocatalysis and biotransformations are processes that convert a starting material (substrate) into a desired product using a biological system. Biocatalysis uses isolated enzymes, either free or immobilized, to catalyze the reaction, while biotransformation uses whole living cells containing the necessary enzyme(s) [18].

The biotransformation of terpenes is of interest because it allows the production of enantiomerically pure flavors and fragrances under mild reaction conditions [19]. Therefore, biotechnology can help replace the natural scent and aromatic ingredients of plants such as lavender, jasmine or ylang-ylang and metabolically modified microbes can produce a variety of natural molecules ranging from patchoulol, linalool, nerolidol, valencene to sclareol through fermentation.

With the help of biotechnological tools, aroma and fragrance molecules can be produced in some cases more economically and in larger quantities, overcoming many of the drawbacks associated with chemical synthesis or plant extraction [20].

### 2.1. Genetic Engineering of Plants for the Production of Terpenoids

Engineering of terpene metabolism in plants is an attractive alternative because of their elaborate biosynthetic potential and the obvious economic benefit of using photosynthesis to drive production [21]. Moreover, another benefit is the less expensive extraction of essential oils: the methods for their extraction, if applied on a large scale, would require little optimization and limited investments, as the methods themselves are already well known on an industrial level. 

Plants can be genetically engineered by means of the introduction of foreign genes or via the modulation of the expression of one or more native genes. Valid pioneering experiments performed mainly on herbaceous plants have paved the way for the genetic manipulation of the odorant trait, highlighting the potential of the expression of heterologous terpene synthase in changing the volatile profile. Interesting results have also been obtained with woody plants and mosses. For example, in 2010, Ohara et al. [22] engineered *Eucalyptus camaldulensis*, a woody plant which is widely used for the production of cellulose for the pulp and paper industries, and essential oils, by means of the expression of a heterologous synthase (the limonene synthase from *Perilla frutescens*, PFLS), in order to increase its limonene content. Similarly in 2014, Zhan et al. [23] engineered the moss *Physcomitrella patens* by means of two heterologous synthases, *Pogostemon cablin* patchoulol synthase (PTS) and *Santalum album* α/β-santalene synthase (STS), respectively to increase patchoulol and α- and β-santalene; the latter is the precursor of α- and β santalol. 

**Figure 1:**
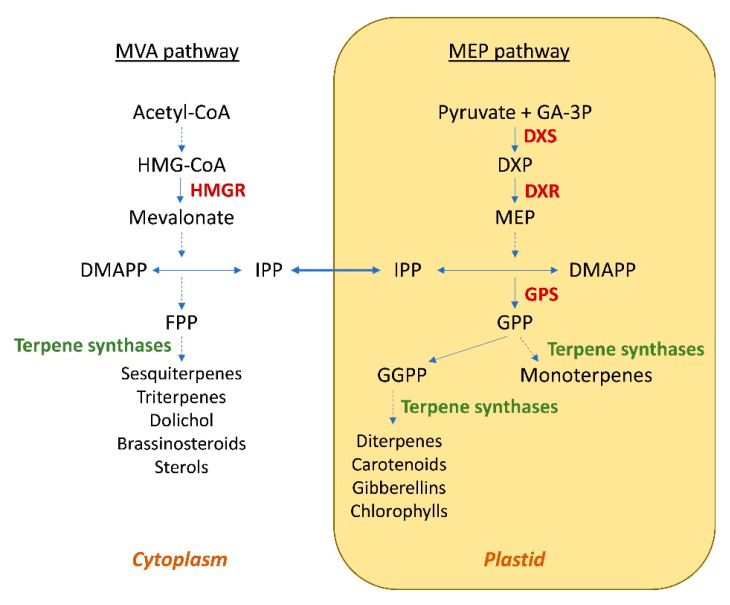
Subcellular organization of the MVA and MEP pathways in plant cells. HMG-CoA—3-hydroxy-3-methylglutaryl coenzyme A; HMGR—3-hydroxy-3-methylglutaryl coenzyme A reductase; DMAPP—dimethylallyl diphosphate; IPP—isopentenyl diphosphate; FPP—farnesyl diphosphate; GA-3P—glyceraldehyde 3-phosphate; DXP—1-deoxy-d-xylulose 5-phosphate; DXS—1-deoxy-d-xylulose 5-phosphate synthase; DXR—1-deoxy-d-xylulose 5-phosphate reductisomerase; MEP—2-*C*-methyl-d-erythritol-4-phosphate; GGPP—geranylgeranyl diphosphate; GPP—geranyl diphosphate; GPS—geranyl diphosphate synthase. Dashed-line arrow indicates multiple steps.

The compartmentalization of the biosynthesis of terpenes in plants was also considered in these studies. In fact, the cytosolic pathway is predominantly responsible for the generation of C_15_-derived terpenes, such as sterols and sesquiterpenes, whereas monoterpenes (C_10_), diterpenes (C_20_) and tetraterpenes (C_40_; e.g., carotenoids) are synthesized via the plastidic pathway [21] (Figure 1). Indeed, it has been observed that the modification of the subcellular localization of terpene synthases can lead to an increase in the production of terpenes.

For this reason, Zhan et al. [23] attempted to re-localize the synthases responsible for the production of sesquiterpenoids patchoulol and α- and β-santalene to the plastids of *P. patens* by adding the transit peptide of the small subunit of the RuBisCO enzyme from Arabidopsis. On the contrary, PFLS [22], like many other monoterpene synthases, is localized in the plastids and already has a plastid localization signal at the N-terminal. Therefore, to evaluate the different levels of expression in the cytosol, a second version of PFLS, lacking the putative signal peptide, was expressed in *E. camaldulensis*.

**Figure 2:**
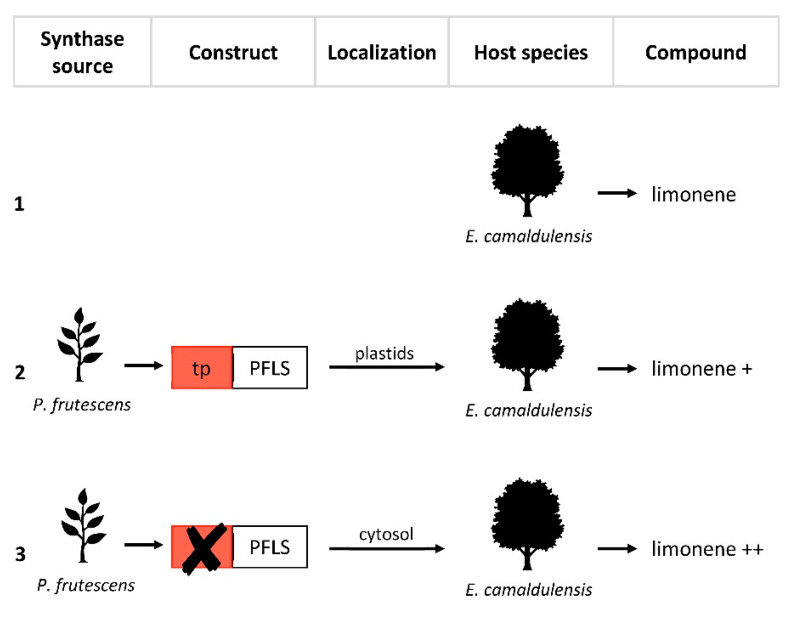
Re-localization approaches of limonene synthase from *Perilla frutescens*. PFLS, *P*. *frutescens* limonene synthase with plastid localization signal at the N-terminal (tp). (**1**) Production of limonene in wild-type *Eucalyptus camaldulensis*. (**2**) Transformation of *E*. *camaldulensis* with PFLS. (**3**) Transformation of *E*. *camaldulensis* with modified PFLS by removal of tp. The “+” symbols indicate an increase in limonene production compared to (**1**).

These approaches showed that cytosolic expression of PFLS allows for a significantly greater (4.5-fold) accumulation of limonene than that found with the native plastid expression (Figure 2, Table 1), suggesting that the cytosolic PFLS could somehow effectively use the cytosolic GPP as a substrate.

**Figure 3:**
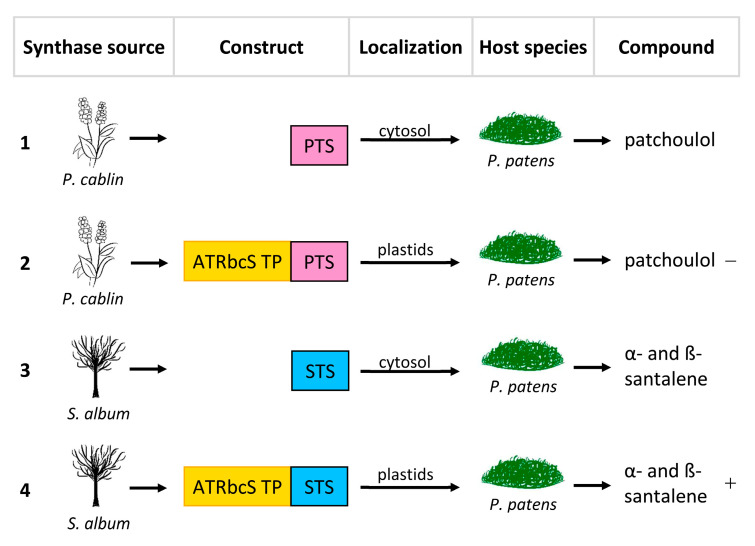
Re-localization approaches of patchoulol synthase from *Pogostemon cablin* (PTS) and α/β-santalene synthase from *Santalum album* (STS). ATRbcsS TP, transit peptide of the small RuBisCO subunit of *Arabidopsis thaliana*. (**1**) Transformation of *Physcomitrella patens* with PTS. (**2**) Transformation of *P*. *patens* with PTS + ATRbcsS TP. (**3**) Transformation of *P*. *patens* with STS. (**4**) Transformation of *P*. *patens* with STS + ATRbcsS TP. The symbols “−” and “+” indicate, respectively a decrease and an increase in production.

The same cannot be said for the plastid targeting of PTS, which showed decidedly lower yields, suggesting that the pool of FPPs in the plastids could be limited, thus confirming how the availability of substrate is fundamental for the correct heterologous expression of a compounds of interest; a solution, therefore, could be to up-regulate the transcription of genes that code for FPP. Finally, plastid targeting of STS resulted in a noticeable increase in α- and β-santalene (Figure 3, Table 1).

Another experiment conducted by Zhang et al. (2020) [24] on a liverwort plant system, which is fast and easy to culture, *Marchantia paleacea*, showed that the production of sesquiterpenoid patchoulol was feasible and had potential. In this experiment, the compartmentalization of FPS and PTS in the chloroplasts and cytoplasm of different transformant plants showed no significant difference in the yield of patchoulol. However, the highest yield of patchoulol (3250 μg/g DW) was achieved in transformant plants with similar transcription levels of FPS and PTS, which was in turn obtained via the introduction of a fusion protein and the co-introduction of individual proteins all equipped with plastid targeting [24]. Thus, it was found that the compatibility of exogenous pathways and plant endogenous pathways is an ideal state for efficient synthesis [24].

Although the aforementioned experiments can be considered successful, the yields obtained using these approaches are still not high enough to compete with the traditional approaches. For example, in the leaves of *P. cablin* (which is the source currently used to obtain patchoulol) the patchoulol content measured in the essential oil [25] is equal to 39% dry weight, corresponding to a quantity of about 10 mg/g dry weight of leaf. This content is about three times higher than that measured in *M. paleacea.*

Another aspect is that in many plants the accumulation or release of terpenes largely depends on the presence of specialized structures, such as glandular trichomes or resin ducts. For example, in nature, limonene is accumulated mainly in the small vesicles located in the flavedo or in the exocarp of citrus fruits, while patchoulol is accumulated in two different cell types: it can be found in the glandular trichomes present on the epidermis of the leaves or in the internal cells of the spongy mesophyll. In this system, the co-expression of an efflux transporter could be effective in excreting essential oil compounds outside the cells, although such monoterpenes-specific transporters are, as far as we know, still unknown. Furthermore, a conceptually attractive approach to enhance the production of terpenes could also be to manipulate the transcription factors thereby increasing the amount of the aforementioned specialized structures.

As we will see, until now fragrance compound yields obtained experimentally through the metabolic engineering of plants have turned out to be significantly lower than those obtained through the traditional methods or with microorganisms (see Section 2.3, “Comparison of plant- and microorganism-based systems”). In fact, as seen, one of the main disadvantages of this approach is the complexity of the biochemistry of terpenes in plants (compartmentalization of the biosynthesis of terpenes in plants, presence or absence of specialized structures, etc.), which makes the time necessary to generate stable gene transformations longer, and consequently, the initial cost higher.

### 2.2. Microbial Terpenoid Production

Although plants are the natural source of terpenoids, recent years have been characterized by a remarkable increase in the production of terpenoids through the microbial route [26]. The use of microbial cell factories in the production of natural fragrance compounds may potentially overcome challenges that might come up not only with the traditional approaches but also with plant genetic engineering approaches. First, microbial biosynthesis allows for industrial-scale production of pure compounds. Second, microbial conversion can save time and costs due to higher yields, faster growth (when compared to plants), and easier product recovery. Finally, microbial production can use abundant, renewable and/or sustainable stocks, such as biological waste (carboxylic acids) or lignocellulosic biomass [27], is independent from climatic conditions and has a higher level of sustainability when compared to chemical production processes; even a rare essential oil can be isolated and produced in large quantities [20].

One of the main advantages of producing fragrant substances from microorganisms is, however, the easier genetic manipulation due to their smaller and less complex genome. In most cases the first strategy is generally to transfer an exogenous terpene synthase into the host microorganism and to perform heterologous expression, while the second focuses on the optimization of the endogenous pathway (for example, via the up-regulation of limiting genes in the pathway and the down-regulation of genes involved in the competitive pathways). Of the two, the first strategy has the main advantage of avoiding possible feedback regulation mechanisms since the metabolites are foreign to the producing host cells.

However, as in the case of plants, it must be considered that the expression of heterologous genes within the host could affect their endogenous metabolism or that, sometimes, some products could generate cytotoxicity. Therefore, in addition to optimizing metabolic pathways and flux, it is also often necessary to provide integrated removal strategies in situ (“In Situ Product Removal”, ISPR). In fact, due to the high volatility of these type of compounds and the possible inhibitory effects on cell growth, it is necessary to remove the product during fermentation through methods, such as gaseous “stripping” or double phase separation, the latter being particularly suitable for higher yields. Furthermore, the choice of a suitable host strain must be considered very carefully, because this also determines the success of developing a cost-effective production process [28]. Studies describing the biocatalysis/biotransformation of terpenes using enzymes, cell extracts and whole cells of bacteria, cyanobacteria, yeasts, microalgae, fungi and plants have been published [19]. Ideal host strains should be well-characterized in terms of their genome sequence and annotation and also be genetically accessible. However, the modification of compounds by plant enzymes, which may be absent in the host, can be a limiting factor in these types of approaches. Most of the complex terpenes of industrial interest are selectively hydroxylated by enzymes belonging to the cytochrome P450 family. From this perspective, *Saccharomyces cerevisiae* is considered a more adequate host for heterologous expression because yeast naturally expresses P450 enzymes that are structurally similar to plant enzymes. However, alternative strategies have also been studied to maximize productivity in *Escherichia coli*, with the most promising focusing on engineering bacterial P450s by mutagenesis to alter their catalytic properties. The validity of this approach is further supported by the observation that bacterial P450s are more stable in prokaryotic systems and tend to have a higher turnover rate when compared to their eukaryotic counterparts [10]. Moreover, to make the production cost-effective, microorganisms should be easily cultivable under laboratory conditions and in production-scale, grow quickly with simple nutrient demands, and feed on low-cost feedstocks [28].

Usually, the most used microorganisms for these types of processes have been model microorganisms *S. cerevisiae* and *E. coli*. In yeasts and bacteria, the existence of two distinct biosynthetic pathways, namely the MVA pathway in yeasts and the MEP pathway in bacteria, has led to the consideration of complementary approaches to generate “cell factories” capable of producing the terpene compounds of interest.

However, in relation to the target molecule, the metabolic pathways and the carbon source to be used, alternative host organisms can exhibit genetic and physiological advantages when compared to *E. coli* or *S*. *cerevisiae*. In particular, the advancing development of -omics technologies and novel metabolic engineering tools for non-coli/non-cerevisiae hosts have made alternative organisms available for microbial cell factory design [28]. For example, in a study [29], whole-cell biotransformation by the yeast *Hyphozyma roseoniger* was exploited, and the metabolites involved were identified and quantified using NMR spectroscopy and LC–MS metabolomics.

There are organisms which can use a wide range of cheap non-sugar carbon sources, for example, *Methylobacterium extorquens* [30] or the yeast *Yarrowia lipolytica.* In fact, approaches similar to the ones conducted on *S. cerevisiae* were conducted on *Y. lipolytica* to produce linalool [31] and α-santalene [32] and the final results demonstrated that *Y. lipolytica* provides a compelling platform for the production of terpene compounds. Using autotrophic microorganisms could also become profitable, since these organisms are able to assimilate nonorganic carbon, such as atmospheric CO_2_ [28]. Some examples are the photoautotrophic cyanobacterium *Synechocystis* sp. [33], the chemolithoautotrophic organism *Cupriavidus necator* [34], or the purple bacterium *Rhodobacter capsulatus*, the latter offering unique physiological properties that are favorable for biosynthesis of hydrophobic terpenes [35]. Other alternative host organisms for terpenoid production are, for example, *Bacillus subtilis* [36] and *Pichia pastoris*, which have been engineered to produce nootkatone, often used in the fragrance sector due to its pleasant grapefruit-like aroma [37,38].

In addition to the beneficial genetic and physiological properties of these alternative organisms, establishing terpenoid production processes with these unusual hosts, although time consuming, might be a way to find a gap in the crowded patent landscape [28]. Nonetheless, the genetic tools available for these fewer known platforms are still behind those developed for baker’s yeast and *E. coli*. Therefore, it could also be an option to transfer useful traits from nonconventional strains, such as tolerance factors, to the genetically well tractable platform hosts, depending on the complexity of the factors to be copied [28]. For example, *E. coli* in its native form can grow well at a temperature range of between 20 and 40 °C. On the other hand, *B. subtilis* can survive at high temperatures due to its spore-forming ability [26].

In this work, we will mainly focus on *E. coli* and *S. cerevisiae*, as they are shown to be the most used for these types of studies.

#### 2.2.1. *Escherichia coli*

Isoprenoid production in bacterial hosts must often deal with the low content of the IPP and DMAPP precursors which are produced by the endogenous MEP pathway. For this reason, bacterial hosts such as *E. coli* have often been engineered to improve IPP and DMAPP synthesis by augmenting bottleneck enzymes of the MEP pathway or introducing a heterologous MVA pathway [39].

As an example, in a recent work [40], the monoterpene limonene was produced by using *E. coli* as a host microorganism, the latter being transformed with a single plasmid on which genes optimized for the MVA pathway were cloned from *S*. *cerevisiae*, together with a geranyl diphosphate synthase and the limonene synthase genes from *Mentha spicata*. These genes were placed under the control of the inducible promoter of the *lac* operon, for the expression of which different inducibility conditions were tested (e.g., concentrations of inducer IPTG). In fact, using an inducible promoter is usually recommended to boost production of heterologous protein only after a sufficient level of biomass has been reached during the initial growth stage: this limits toxicity and growth interference of inducer and the introduced heterologous functions.

Further observations were made for the production of limonene in *E. coli* [40]. First, it was noted that combination of glycerol and lactose (over glucose and IPTG, respectively), has shown beneficial effect on cell viability and productivity of recombinant proteins. It was also noted that an anaerobic environment could decrease the toxicity of limonene hydroperoxide, a common oxidation product that is formed spontaneously in aerobic environments. Lastly, it was noted that in addition to the selection and optimization of the production system, it might be necessary to provide ISPR.

The same approach of heterologous expression of terpene synthases and the improvement of the MVA pathway was used for the production of sclareol, a diterpenol which is widely used as a starting material for the synthesis of fragrant molecules with ambergris notes. Ambergris is a waxy excretion product from sperm whales (*Physeter macrocephalus* L.) which has been used since ancient times as a valued agent in the formulation of perfumes for its pleasant, sweet and earthy scent. Nowadays, ambergris odorants are produced via synthetic or semisynthetic routes [41]. An emblematic example of these type of compounds is Ambrox™ (trade name of Firmenich International SA), a key olfactory component and the most appreciated substitute of ambergris [42]. Ambrox™ is the commercial enantiomerically pure compound equal to the natural one, that is (−)-ambrofuran. Given the scarce availability of the natural source and to avoid running into ethical, economic and supply related problems, valid alternatives have been identified for the synthesis of these type of compounds. Among the alternatives, the synthesis of ambrofuran from sclareol, co-produced in the production of *Salvia sclarea* essential oil, has been shown to be very successful [41]. However, due to the stable and moderate consumption of *S. sclarea* essential oil and the increasing use of ambrofuran, this coupled production process is not meeting the demand.

Therefore, the search for an alternative production route has been the focus of many scientists. For example, Schalk et al. (2012) [42] attempted a biotechnological approach to produce sclareol using *E. coli* as a “cell factory”. The work began with the identification of the two diterpene synthases (DTS) which are responsible for the synthesis of sclareol in *S. sclarea* and was followed by a metabolic engineering approach that involved the expression of a heterologous MVA pathway in the host. Once again, the use of an inducible promoter was exploited, different cultivation conditions were tested and an ISPR strategy was adopted. The results of this study led to the conclusion that because of the high economic value of sclareol, a biotechnological process aimed at the production of this molecule using a “cell factory” could provide a valid alternative or a valid complement both to chemical production and to natural production. Moreover, further bioconversion of sclareol in intermediates (e.g., ambradiol) to shorten the semisynthetic route to ambrofuran was shown to be achievable by whole-cell biotransformation systems [29,41].

Similar conclusions have been drawn from works conducted on *E. coli* addressed in the production of some of the sesquiterpenoids that are abundantly used in the perfume sector, for example santalol and (−)-patchoulol [43] (approaches and results summarized in Table 2). In 2015 [8], a challenging perspective was proposed to tailor enzymes, such as santalene synthase and the hydroxylating enzyme system cytochrome P450 monooxygenase/NADPH-dependent cytochrome P450 reductase (CYP76Fs/CPR), with more efficiency and specificity using advanced protein engineering (e.g., combinatorial mutations generation combined with directed evolution, mutagenesis driven by computational structure predictions of transition state complex, membrane–anchor replacement, and bacterial CYP mutagenesis–refinement).

Recently, Wang et al. (2021) [44] and Zhang et al. (2022) [45] focused on the production of the α-santalol precursor α-santalene, using *E. coli* as host. The *E. coli* gene encoding FPP synthase (*Isp*A) and a plant (*Clausena lansium*) α-santalene synthase gene (*sts*) were combined into a single operon, and associated with a heterologous MVA module both under an IPTG-inducible promoter [44]. On this synthetic system, different combinatorial set of ribosome binding sites were explored to balance expression of coded proteins and to improve the isoprenyl diphosphate production and the synthesis of α-santalene in *E. coli*, reaching a titer of 412 mg/L in a flask culture [44]. In a similar synthetic strategy [45], the engineering of the sesquiterpene biosynthesis pathway to increase α-santalene production in *E. coli* was carried out by screening different FPP synthases and mutagenesis-generated santalene synthase variants to amplify the flux toward farnesyl diphosphate precursor and to improve enzyme efficiency and to tailor substrate specificity in last steps of α-santalene synthesis. The final titer reached 2916 mg/L achieved under fed-batch fermentation (1272 mg/L, in flask culture) [45]. Both studies suggest that *E. coli* is a promising alternative for α-santalene synthesis, providing practical suggestions for terpenoid production through gene and protein engineering. The finalization of the optimized pathways to the synthesis of santalol depends on the efforts needed to express an hydroxylating enzyme system in the *E. coli* host [46], on the engagement of an external biocatalysis or on mixed hosts-based biotransformation strategies.

As we can see from the results summarized in Table 2, to date the expression of a heterologous terpene synthase associated with the introduction of a heterologous mevalonate pathway into *E. coli* seems to be the most promising way to generate a microbial platform for the production of terpene compounds. However, some aspects remain to be improved: the effective use of carbon/sugar sources, the redirection of the host metabolism towards an effective production of the compound of interest (i.e., without compromising the vitality of the host itself), modification of compounds mediated by plant enzymes absent in the host, etc.

**Table 2:** Types of approaches discussed for the microbial production of the selected compounds.

Compound	Host	Approach ^a^	Titer ^b^	Ref.
Limonene	*E. coli*	Introduction of the *Mentha spicata* LS; heterologous expression of MVA pathway	3.63 g/L 7.3 g/L ^c^	[40]
Sclareol	*E. coli*	Introduction of the *S. sclarea* DTSs; heterologous expression of MVA pathway	1.46 g/L	[42]
Santalene	*E. coli*	Overexpression of *E. coli* FPPS (IspA); introduction of plant (*Clausena lansium*) STS; optimization of RBSs; heterologous expression of MVA pathway; removal of competitive indole synthesis by *tna*A deletion	0.60 g/L	[44]
	*E. coli*	FPPSs screening to introduce the selected *S. cerevisiae* mutated FPPS (Erg20^F96W^); tailored mutagenesis of *C. lansium* STS to introduce the selected STS variant (STS^S533A^) and fusion to a solubilization enhancing tag; heterologous expression of MVA pathway	2.92 g/L	[45]
α-santalol β-santalol	*S. cerevisiae*	Introduction of *S. album* CYP (CYP736A167), CPR (CPR2) and STS; manipulation of MVA pathway for the use of galactose-based regulation system	1.18 g/L	[47]
Patchoulol	*E. coli*	Introduction of the *P. cablin* PTS; heterologous expression of MVA pathway	0.040 g/L	[43]
Patchoulol	*S. cerevisiae*	Fusion of FPPS (Erg20) and *P. cablin* PTS to increase the utilization of the FPP precursor; manipulation of MVA pathway to enhance its flux to FPP by overexpressing HMGR (tHMG1), IDI (IDI1), and UPC2-1, and by repressing competitive steps	0.47 g/L	[9]
Geraniol	*S. cerevisiae*	Introduction of *Ocimum basilicum* codon-optimized GS; manipulation of MVA pathway to funnel it to GPP production by overexpressing HMGR (tHMG1), IDI (IDI1), MAF1 and mutated FPPS (Erg20^K197G^) catalytic domains	0.036 g/L	[48]
Linalool	*S. cerevisiae*	Introduction of *Mentha citrata* LIS variant (t67OMcLIS^E343D/E352H^) generated by directed evolution ^d^; overexpression of MVA pathway and mutated FPPS (Erg20^F96W/N127W^)	0.053 g/L	[49]

^a^ Description of features of the culture procedure are not included; ^b^ expressed in grams per liter of culture; ^c^ referred to the organic phase of the culture; ^d^ directed evolution is a mutagenesis approach that allows a positive selection of variants with improved catalytic (or other desired feature) performance. Abbreviations: LS—limonene synthase; MVA—mevalonate; DTS—diterpene synthase; PTS—patchoulol synthase; FPPS—farnesyl diphosphate synthase; GPP—geranyl diphosphate; FPP—farnesyl diphosphate; STS—santalene synthase; RBS—ribosome binding site; GS—geraniol synthase; MAF1—negative regulator of tRNA isopentenyltransferase; IDI—isopentenyl diphosphate isomerase; HMGR—3-hydroxyl-3-methylglutaryl-CoA reductase; LIS—linalool synthase; CYP—cytochrome P450; CPR—CYP reductase.

#### 2.2.2. *Saccharomyces cerevisiae*

Despite, the evident cell-structural and metabolic differences with respect to plant cells, yeasts (particularly *S. cerevisiae*) were thought to have some preferable features over prokaryote-based systems to be widely used as a host to produce aromatic compounds [50]. Unlike prokaryotes, yeasts have organelles, which provide various compartments and environments within which the biosynthesis of terpenes can take place. Furthermore, as a model eukaryotic system, *S. cerevisiae* offers many advantages especially in terms of the growth rate, the extent of engineering tools applicable to it, bio-security (*S. cerevisiae* is Generally Recognized As Safe—GRAS—according to the United States Food and Drug Administration designation), robustness (when used at industrial levels) and easy genetic manipulation. However, yields may be lower mainly due to the weak flow of the MVA pathway in yeasts, suggesting that the poor availability of precursors (particularly GPP) from the MVA pathway is a very important factor to be taken into consideration when it is desired to obtain an increased biosynthesis of terpenes in this type of host. In fact, the general metabolic engineering strategies for the synthesis of terpene compounds in *S. cerevisiae* concern enhancing the flow of the MVA pathway by overexpressing the key genes; downregulating the competitive pathways by replacing the native promoters with inducible ones; knocking-out or inhibiting some negative regulators; and strengthening terpene synthases or other enzymes associated with them, through overexpression or protein engineering.

In this regard, Liu et al. (2013) [48] and Zhou et al. (2020) [51] tried to overcome the problem by manipulating three genes that have been shown to be related to an increase in the flow of the MVA pathway in order to produce geraniol and linalool, two monoterpenes which are widely used as heart notes in the perfume industry due to their pleasant rose and floral/spicy smell, respectively. The three genes in question are *tHMG*1, *IDI*1 and *ERG*20. The *tHMG*1 gene codes for a truncated and deregulated version of yeast HMG1 that preserves the hydroxymethylglutaryl-CoA (HMG-CoA) reductase (HMGR) activity. HMG1 is one of the two HMGR isozymes present in yeast having different regulatory features [52]. The yeast *IDI*1 gene encodes an isomerase that catalyzes the isomerization of IPP to DMAPP and that was thought to adjust their relative abundance, thus favoring the production of GPP and therefore of the two monoterpenes of interest. Lastly, unlike plants *S. cerevisiae* lacks a GPP-specific synthase, so both GPP and FPP are synthesized by the same FPPS enzyme, encoded by *ERG*20. The greater accumulation of both geraniol and linalool which followed the expression of the above-mentioned genes, coupled with the introduction of a specific exogenous terpene synthase demonstrates the important role that the availability of GPP plays in the production of both monoterpenes.

Additionally, in the case of the engineering of *S. cerevisiae* for the production of sesquiterpene compounds, the overexpression of key genes for the MVA pathway allowed to obtain satisfactory yields. For example, in the work of Ma et al. (2019) [9] for the production of patchoulol, *ERG*20 (encoding FPPS) and PTS (encoding patchoulol synthase) were fused to generate a bifunctional FPTS protein which was meant to be expressed in yeast, in order to make the use of the FPP precursor more efficient and therefore to increase the production of patchoulol. Furthermore, limiting genes *tHMG*1, *IDI*1 and *upc2*-1 (encoding an activated allele of the transcription factor UPC2 and UPC2-1, which is involved in increasing the expression of genes for the utilization of sterols and the MVA pathway) were integrated into the genome to improve the flow of the MVA pathway. In another study, Zha et al. (2020) [47] placed the expression of genes which are related to the biosynthesis of α- and β-santalol under the control of GAL promoters (P_GAL_), thus allowing to exploit the GAL regulation system for the biosynthesis itself.

**Figure 4:**
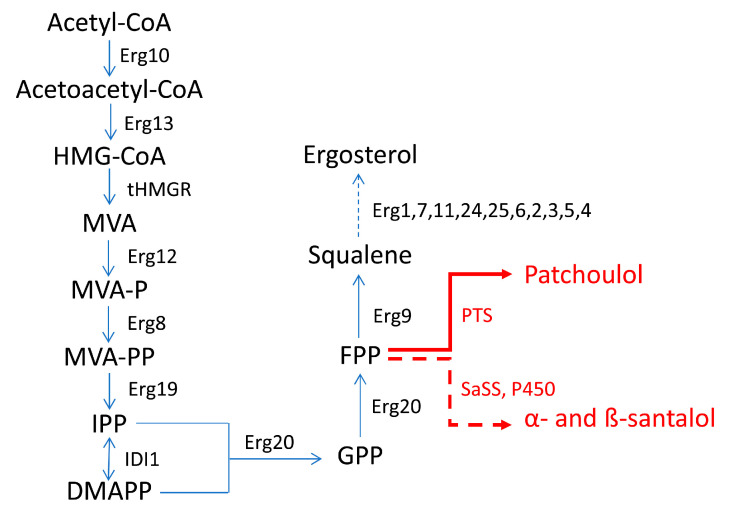
Biosynthetic pathway to produce patchoulol and α- and β-santalol in *S. cerevisiae* in the works of Zha et al. (2020) [47] and Ma et al. (2019) [9]. Erg10—acetyl-CoA C-acetyltransferase; Erg13—hydroxymethylglutaryl-CoA synthase; tHMGR—truncated 3-hydroxyl-3-methylglutaryl-CoA reductase; Erg12—mevalonate kinase; MVA—mevalonate; MVA-P—mevalonate phosphate; MVA-PP—mevalonate diphosphate; Erg8—phosphomevalonate kinase; Erg19—diphosphomevalonate decarboxylase; IDI1—isopentenyl diphosphate δ-isomerase; Erg20—farnesyl diphosphate synthase; Erg9—squalene synthase; SaSS—α-santalene synthase from *S. album*; PTS—patchoulol synthase; P450—enzymes of the cytochrome P450 family (CYPs). Dashed-line arrow indicates multiple steps.

In both works [9,47], *ERG*9 (encoding a squalene synthase) was placed under the control of the glucose-inducible promoter P_HXT1_ (promoter of the *HXT*1 gene) to reduce the metabolic flux from FPP to ergosterol. In fact, FPP is a common precursor of the synthesis of either the latter and of α- and β-santalol and patchoulol (Figure 4). In *S. cerevisiae*, the MVA pathway is the only pathway for the biosynthesis of isoprenoid precursors and originally leads to the formation of ergosterol as the main product in yeast cells. In the case of ergosterol biosynthesis, FPP is converted into squalene by the squalene synthase (encoded by the *ERG*9 gene). Squalene can be converted into ergosterol, which is essential for yeast growth. Since yeast cells are unable to assimilate exogenous ergosterol during aerobic growth, the *ERG*9 gene cannot be erased completely. To increase the availability of FPP for the synthesis of patchoulol and α- and β-santalol, the native promoter of the *ERG*9 gene was then replaced with a P_HXT1_ promoter.

Overall, the results of these works are summarized in Table 2. As we can see, it was once again highlighted how, sometimes, the overexpression of biosynthetic enzymes and key regulatory proteins may not be enough to increase the yields of the target compounds, and how often the latter can be increased through the use of inducible promoters. Furthermore, in the case of sesquiterpene compounds, the greatest limit seems to be associated with the production of ergosterol, which is essential for the vitality of yeast cells. Further approaches which could improve the flow to the MVA pathway without compromising the viability/survival of the yeast cells themselves remain to be discovered. Such approaches could help to obtain greater biomass, thus also generating greater yields of the compounds of interest.

### 2.3. Comparison of Plant- and Microorganism-Based Systems

A general analysis of the selected cases, among the comparable ones, made by considering only the titer of the produced compound as parameter, reveals a probable superiority of microbial-based biotechnological approaches with respect to the plant one. For example, the titer of limonene obtained using microbial means is approximately 2200 times greater than the same obtained following genetic engineering interventions of *E. camaldulensis*. Similarly, the titers of patchoulol, santalene and santalols obtained using microbial means are, respectively about 96 and more than 20,000 times greater than those obtained following genetic engineering interventions of *P. patens* (Table 3). It must be noted that to evaluate the economic feasibility, a more in depth analysis is needed case by case by considering not only the titer parameter but also direct and indirect costs associated with these biotechnological approaches, such as those regarding biomass (plant or microorganism) growth and the final isolation of the compound. Actually, the presence of marketed raw materials for the perfume industry (e.g., Ambrox™, Ambrofix™, Clearwood™; [25,28,53]) produced employing microbial biotransformation proves the viability of this approach.

## 3. Concluding Remarks

In recent decades, biotechnology has played an increasingly important role in the fragrance industry, enabling the sustainable and high-quality production of fragrance ingredients, such as terpenoids. The advances in the field of biochemistry and gene manipulation techniques and the use of metabolic engineering interventions in plants and microorganisms have led to a deeper understanding of the biosynthesis of terpenes, allowing the development of new biotechnological pathways for the production of compounds traditionally extracted from natural resources.

Modifying the volatile profile of plants has proven difficult through conventional breeding technologies alone. However, metabolic engineering interventions, such as the introduction of specific synthase genes and the modulation of the mevalonate pathway, have been successful in altering terpene metabolism. Despite the economic convenience of using plants as platforms for the production of terpene compounds commonly exploited in the fragrance sector, the complexity of biosynthetic pathways and the dependence on specialized structures, such as glandular trichomes or resin ducts, limit the efficiency of production. Re-localizing terpene synthases responsible for the production of certain terpenoids to the plastids has shown promise in increasing their production, although yields still need to be improved to compete with traditional approaches.

Microbial biosynthesis provides numerous advantages over traditional methods, including cost and time savings. Biotransformation can occur in water and at non-extreme temperatures with high enantioselectivity, making it more sustainable and energy-efficient than the chemical synthesis or the extraction from natural resources. However, when considering the economic and logistical aspects of production, it is essential to carefully select an appropriate host strain. Although genetic tools for nonconventional strains are not as advanced as those for model microorganisms, such as *S. cerevisiae* and *E. coli*, alternative host organisms with favorable genetic and physiological traits need to be explored to achieve a cost-effective production process.

Despite the potential of microbial-based approaches to address supply difficulties and demand for sustainable ingredients and processes, scaling up production can present challenges, such as yield optimization, contamination control, and regulatory compliance. Nevertheless, by addressing these issues and developing efficient production processes, biotechnology solutions can help meet the growing demand for sustainable and high-quality fragrance ingredients. Recent successful launches of new biotechnologically produced ingredients in the perfume industry indicate that biotechnological processes will continue to gain popularity due to their improved sustainability, higher quality, and reduced cost.

## Figures and Tables

**Table 1:** Types of approaches discussed for the plant production of the selected compounds.

Compound	Host	Approach	Titer ^a^	Ref.
Limonene	*Eucalyptus camaldulensis*	Native wild-type (not engineered)	73 µg ⁄g FW ^b^	[22]
		Introduction of *Perilla frutescens* LS	190 µg ⁄g FW ^b^	[22]
		Introduction of *P. frutescens* tp-deprived LS	327 µg/g FW ^b^	[22]
Patchoulol	*Marchantia paleacea*	Introduction of codon-optimized *Gallus gallus* FPPS and *P. cablin* PTS, both equipped with tp and driven by the 35S promoter	3250 µg/g DW ^c^	[24]
	*Physcomitrella patens*	Introduction of *P. cablin* PTS and *S. cerevisiae* truncated HMGR	1340 µg/g DW	[23]
		Introduction of *P. cablin* PTS	830 µg/g DW	[23]
		Introduction of *P. cablin* PTS equipped with tp	20 µg/g DW	[23]
α-santalene β-santalene	*Physcomitrella patens*	Introduction of *S. album* STS	n.d.	[23]
		Introduction of *S. album* STS and *S. cerevisiae* truncated HMGR	α: 22 µg/g DW β: 20 µg/g DW	[23]
		Introduction of *S. album* STS equipped with tp	α: 39 µg/g DW β: 35 µg/g DW	[23]

^a^ Expressed in µg per gram of plant material; ^b^ mature leaves; ^c^ thalli. Abbreviations: LS—limonene synthase; tp—transit peptide; FW—fresh weight; DW—dry weight; PTS—patchoulol synthase; FPPS—farnesyl diphosphate synthase; HMGR—3-hydroxyl-3-methylglutaryl-CoA reductase; STS—santalene synthase; n.d.—not detectable.

**Table 3:** Some biotechnological approaches mentioned compared for the yields of terpene compounds commonly used in the production of perfumes.

Compound	Host	Titer ^a^
Limonene	*E. camaldulensis*	(327 mg/kg FW) 0.33 mg/g FW [22]
	*E. coli*	3630 mg/L (726 mg/g FW) [40]
Patchoulol	*M. paleacea*	(325 mg/kg FW) 3.25 mg/g DW [24]
	*S. cerevisiae*	470 mg/L (313 mg/g DW) [9]
α-santalene, santalols	*P. patens*	(3.9 mg/kg FW) ^b^ 0.039 mg/g DW ^b^ [23]
	*S. cerevisiae*	1180 mg/L (787 mg/g DW) [47]
	*E. coli*	2920 mg/L ^b^ (5840 mg/g DW) ^b^ [45]

^a^ Expressed in mg per liter of culture for microbial hosts and in mg per gram of plant material in case of plant hosts; in brackets are calculated values (percentage of water considered in fresh biomass, 90%; biomass yield considered in culture, 5 g/L for *E. coli* and 15 g/L for *S. cerevisiae*). ^b^ α-santalene.

## Data Availability

Not applicable.
